# Generation of Peanut Drought Tolerant Plants by Pingyangmycin-Mediated *In Vitro* Mutagenesis and Hydroxyproline-Resistance Screening

**DOI:** 10.1371/journal.pone.0119240

**Published:** 2015-03-31

**Authors:** Jiongming Sui, Ya Wang, Peng Wang, Lixian Qiao, Shimeng Sun, Xiaohui Hu, Jing Chen, Jingshan Wang

**Affiliations:** 1 College of Life Science, Qingdao Agricultural University/ Key Laboratory of Plant Biotechnology in Universities of Shandong/ Key Laboratory of Qingdao Major Crop Germplasm Resource Innovation and Application, Qingdao, China; 2 Peanut Research Institute, Shandong Academy of Agricultural Sciences, Qingdao, China; Institute of Genetics and Developmental Biology, Chinese Academy of Sciences, CHINA

## Abstract

In order to enlarge the potential resources of drought-tolerant peanuts, we conducted *in vitro* mutagenesis with Pingyangmycin (PYM) as the mutagen as well as directed screening on a medium supplemented with Hydroxyproline (HYP). After being extracted from mature seeds (cv. Huayu 20), the embryonic leaflets were cultured on somatic embryogenesis-induction medium with 4 mg/L PYM and the generated embryos were successively transferred to a germination medium with 4 and then 8 mmol/L HYP to screen HYP-tolerant plantlets. After that, these plantlets were grafted and transplanted to the experimental field. In the next generation, all seeds were sown in the field, and phenotype variation and trait segregation can be observed in most of the offspring (M_2_ generation). The M_3_ generation individuals were subjected to drought stress at the seedling stages. The activities of SOD and POD were substantially increased in eight offspring of 11 HYP-tolerant, regenerated plants than in their mutagenic parents. To determine the correlation between mutant phenotypes and genomic modification, we carried out a comparison of the DNA polymorphisms between the mutagenic parents and 13 M_3_ generation individuals from different HYP-tolerant, regenerated plants with SSR primers. Results showed that most mutants and parent plants had signs of polymorphisms. Under drought stress, some M_3_ generation individuals of 10 original HYP-tolerant, regenerated plants produced more pods than the mutagenic parent; twenty individuals among them produced >60 g pods/plant. M_4_-generation seeds were tested for quality characteristics by Near Infrared Spectroscopy (NIS) and nine individuals with higher protein content (>30%) and 21 individuals with higher oil content (>58%) were screened. We concluded that the use of PYM-based *in vitro* mutagenesis in combination with directed screening with HYP is effective for the creation of potential drought-tolerant mutants of peanut.

## Introduction

As one of the key oil crops in China, peanut mainly grows in arid and semiarid areas where biotic and abiotic stresses pose great challenges to its growth and yield [1]. Although there have been efforts to obtain high-yield and stress-resistant peanut cultivars through traditional breeding methods, few breakthroughs have been made due to the lack of stress-resistant germplasm. Compared with conventional breeding approaches, mutation-assisted breeding has proven to be more effective in developing cultivars with high resistance to various abiotic stresses [2–6].

It usually takes a substantial amount of labor and time to screen the huge quantities of mutants generated via mutation technology. However, through the combination of *in vitro* mutation and tissue culture, the labor and time consumption can be effectively decreased and researchers could rapidly isolate variants with desired agronomic traits. Besides, this approach can also reduce chimerism, and mutants obtained can be directly used to breed new crop varieties [5, 7–10].

Although *in vitro* mutagenesis has been reported, only a few mutants have been reported by now. Venkatachalam et al. (1997) treated peanut calli with ethyl methane sulfonate and gamma radiation and obtained a few disease-resistant genotypes by screening on a medium with culture filtrate of the plant-pathogenic fungus *Phaeoisariopsis personata* [11]. Another study on *in vitro* mutagenesis was performed by Muthusamy et al. (2007) who treated peanut’s embryonic calli with gamma radiation or ethyl methane sulfonate to induce somatic embryogenesis on culture medium [12]. Results showed that regenerated plants diverged from their parent plants in many aspects, including leaf shape, pod number, and pod weight per plant. However, studies on *in vitro* mutagenesis combined with HYP screening for drought-tolerant peanuts have not been reported to date.

In our previous experiment, we have described the effect of PYM as a mutagen on the formation of somatic embryos in different peanut varieties [10]. In the present study, we combined *in vitro* mutagenesis with PYM and screening with HYP, which functioned as an osmoticum, to obtain potentially drought-tolerant mutants of peanut.

## Materials and Methods

### Peanut cultivars and tissue culture media and conditions

In this research, Huayu 20, a drought-sensitive peanut cultivar usually grown in northern China, was used as the experimental subject. The major components of the somatic induction medium included MS basal salts, B_5_ vitamins, 3% sucrose, 0.8% agar, and 10 mg/L 2,4-D, while the mutagenesis medium was an induction medium supplemented with 4 mg/L PYM [13]. In contrast, the germination medium was a mixture of MS basal salts, B_5_ vitamins, 3% sucrose, 0.8% agar, and 4 mg/L 6-benzyl aminopurine, while the screening medium was germination medium supplemented with various concentrations of HYP. Regarding the pH value of all the media and *in vitro* cultures, we kept it the same as that in our previous report [13].

### Determination of the optimal concentrations of HYP for selection pressure

After surface-disinfestation and immersion in sterile water for 12 hours, the embryos without cotyledons were dissected. For somatic embryogenesis, the embryonic leaflets (explants) were cultured on induction medium for 4 weeks after they were removed from the embryos.

The optimal concentration of HYP for screening was determined by two steps: the first step occurred in embryo generation period while the second took place during plantlet regeneration from embryos. In the first step, germination media with different HYP concentrations (0, 2, 4, 6, or 8 mmol/L) were used to nurture the somatic embryos generated on the induction medium; by observing the response of the somatic embryos, we could determine the lethal and screening concentrations of HYP in culturing somatic embryos. In the second step, we transferred the somatic embryos that germinated on HYP-free germination medium to the same medium that contained different HYP proportions (0, 4, 6, 8, 10, 12 mmol/L). Four weeks later, based on the response of the germinated somatic embryos, we determined the lethal and screening HYP concentrations for the growth of regenerated plantlets. Each determination included three replications and each replication included 50 explants.

### Mutagenesis, selection, and grafting of HYP-tolerant, regenerated plantlets

Based on the optimal HYP concentration determined in the previous section, we conducted mutagenesis and selection. To induce somatic embryogenesis and mutagenesis, we cultured embryonic leaflets for 4 weeks on mutagenesis medium. After that, the surviving somatic embryos were transferred to the screening medium with 4 mmol/L HYP, the optimal screening concentration for embryo germination (see Results). Then, after growth recovery for 4 weeks on HYP-free fresh germination medium, these embryos were placed on screening medium with 8mmol/L HYP (the optimum screening concentration for plantlet regeneration) to undergo selection (see Results). They were cultured on such medium for 3 weeks. The surviving regenerated plantlets were resected from their bases when they grew to as high as 2 cm and then grafted onto rootstocks as scions. The grafted plantlets were cultured for 2 days and subsequently transferred to pots for acclimation in a greenhouse at 24±2°C [14].

After growth for 3 weeks in greenhouse, these grafted plants were transplanted into the experimental field of Qingdao Agricultural University. That experimental field is typical of aquic soil with a pH value of 6.8. In harvest time, all seeds (M_2_ generation) produced by the regenerated plants were collected and sown into the field in the next year (2012).

In 2012, the phenotypes of individual M_2_ plants were recorded throughout the growing season, and all seeds of the M_3_ generation were collected.

### Measurement of Superoxide dismutase (SOD) and peroxidase (POD) activities

The mature and plump seeds of 11 M2 generation individuals from different HYP-tolerant, regenerated plantlets and similar seeds of their mutagenic parents were sown in pots containing a mixture of soil:sand (2:1) in the greenhouse. The seeds and seedlings were watered regularly until 10 days sowing, at which time water was withheld to subject the plants to drought stress. After water had been withheld for 20 days, a 0.5-g quantity of fresh leaves was collected from each plant and was used to measure the activity of SOD based on the inhibition of nitroblue tetrazolium reduction; the quantity of enzyme extract that inhibited 50% of the reduction was considered to be equivalent to one unit of activity. POD activity was measured by the guaiacol colorimetric method [15, 16]. All enzyme activities were calculated on the basis of fresh weight (FW), and each determination included three replications. Data were analyzed using the SPSS program and the Duncan's multiple range test.

### DNA extraction, purification, and PCR amplification

Young leaves from 3-week-old plants of mutagenic parent and those from 13 M3 generation individuals were taken as samples (one plant for each regenerated plant). With the help of CTAB method as described by Milla et al. [17], their DNA was isolated and the corresponding DNA polymorphism was analyzed through SSR pairs described previously [18].

In SSR analysis, the reaction mixture (10 μl) was composed of 1.0 μl of 10×PCR buffer (2 mM of MgCl_2_), 20 ng of DNA template, 200 μM of each dNTP, 0.4 μM of each primer, and 0.2 units of Taq DNA polymerase. As depicted previously in the study of Qiao et al. [19], sample amplification, polyacrylamide gel electrophoresis and gel staining were carried out in this analysis.

### Measurement of peanut production of M_3_-generation individuals

In 2013, M_3_ seeds obtained from the individual plants in 2012 were sown in the field, and seeds of the mutagenic parents were sown as the controls. The seeds were planted in ridges that were 90 cm containing two lines apart with 17 cm between adjacent seeds. The seedlings were mulched and were watered according to standard practice until 50 days before harvest, at which time water was withheld to impose drought stress. All pods of individual plants were collected and weighted after harvest and sun drying.

### Measurement of quality characters of M_4_-generation seeds

M_4_-generation seeds of 150 individuals from 11 different HYP-tolerant, regenerated plantlets were tested for quality by Near Infrared Spectroscopy calibration model created by Wang [20]. Each line selected one individual and each determination included two replications. Data obtained were analyzed using the SPSS program.

## Results

### The optimal HYP concentration for selecting for HYP tolerance

As described in the Methods, the optimal HYP concentration for selecting HYP-tolerant mutants from the regenerated somatic embryos was determined by two steps: first, the concentration in the period of embryo generation and initial growth was determined; second, the optimal concentration during plantlet regeneration was determined. In the first step, the somatic embryos that had formed on the induction medium were transferred to and cultured on germination media containing HYP at 0, 2, 4, 6, and 8 mmol/L, respectively. Four weeks later, it was found that all somatic embryos on the medium containing 6 mmol/L HYP were dead. It was also found that some embryos on the medium with 4mmol/L HYP survived although severe growth retardation had been caused. Therefore, it was determined that 4 mmol/L was the screening concentration for the germination of embryos which were transferred from the induction medium to the germination medium. In the second step, embryos that germinated on the HYP-free germination medium were transferred to and cultured on screening media with a HYP concentration of 0, 4, 6, 8, 10, and 12 mmol/L respectively. Four weeks later, the survival rate of these embryos was evaluated. In terms of the evaluation results, 10mmol/L was deemed as the lethal concentration while 8mmol/L was selected as the concentration to be used for the screening of germinated embryos that survived the first step.

### Mutagenesis, selection, and grafting of HYP-tolerant, regenerated plantlets and their growth in the field

**Fig 1:**
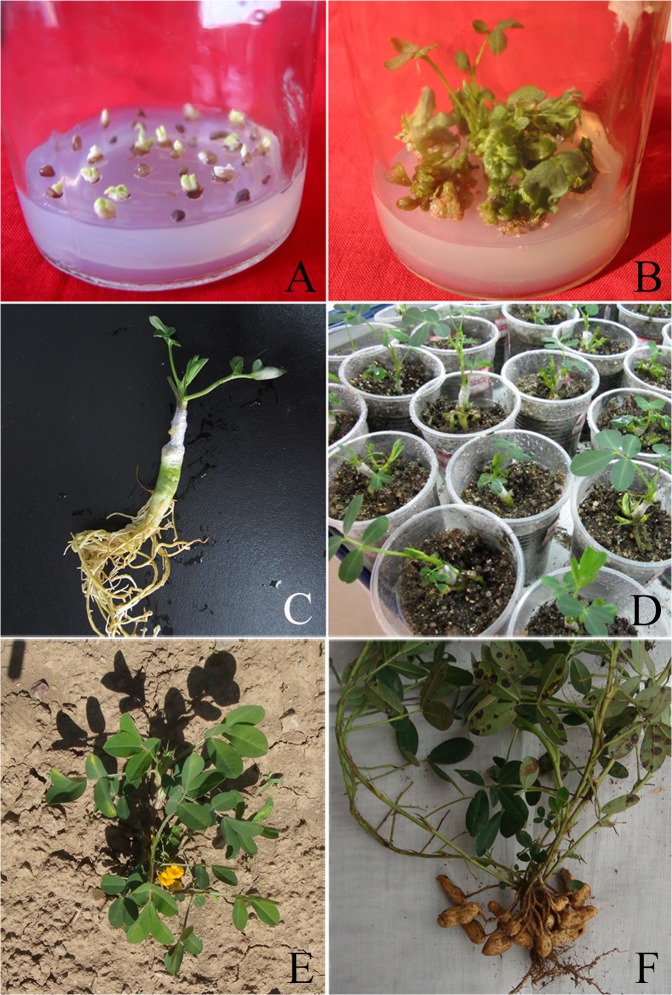
*In vitro* mutagenesis, regeneration of HYP-tolerant plantlets, grafting, and production of pods by grafted plants of Huayu 20. a The somatic embryos formed on the mutagenesis medium (induction medium plus 4 mg/L PYM); b Buds formed after screening on germination medium containing 4 mg/L and then 8 mmol/L HYP; c-d Grafted HYP-tolerant plantlets (the scions are from HYP-tolerant somatic embryos, and the rootstock is cultivar Huayu 23); e An HYP-tolerant plant growing in the field; f An HYP-tolerant plant of the M_1_ generation that produced pods (the M_2_ generation) in the field.

Ten days after being cultured on mutagenesis medium (equivalent to the embryo induction medium plus 4 mg/L PYM), the embryonic leaflets which served as explants started to form ivory calli. After another 3–4 weeks, these explants began to display different growth tendencies: some gradually became brown, while others formed somatic embryos (Fig. 1A). To select HYP-tolerant mutants, the somatic embryos that had taken shape were transferred to the screening medium supplemented with 4 mmol/L HYP. Observation after 4 weeks revealed that most embryos didn’t survive the HYP stress. For the surviving embryos, a HYP-free germination medium was prepared to culture them and help them recover from the HYP stress. Gradually, these surviving embryos began to form a mass of green compact calli. Three weeks later, we transferred them to a screening medium containing 8 mmol/L HYP until plant regeneration took place (Fig. 1B).

When the HYP-tolerant, regenerated plantlets reached 2 cm, their tops were removed and aseptically grafted to the rootstocks of Huayu 23 (Fig. 1C) as scions. After a period of acclimation in the greenhouse (Fig. 1D), these grafted plants were then transplanted to field in 2011 (Fig. 1E). In harvest time, altogether thirteen of them [no. 31–43] produced pods (Fig. 1F).

### Segregation and variation of offspring (M_2_ generation) of the HYP-tolerant, regenerated plantlets

The seeds harvested from the M_1_ generation of the HYP-tolerant, regenerated plants were sown in the field; seeds from Huayu 20 were sown as the controls. The plants (M_2_) were observed for growth, development, and agronomic traits. The offspring of 11 of the 13 original regenerated plants significantly differed from their mutagenic parents in vigor, growth habit, flowering habit, pod shape, and seed coat color.

**Fig 2:**
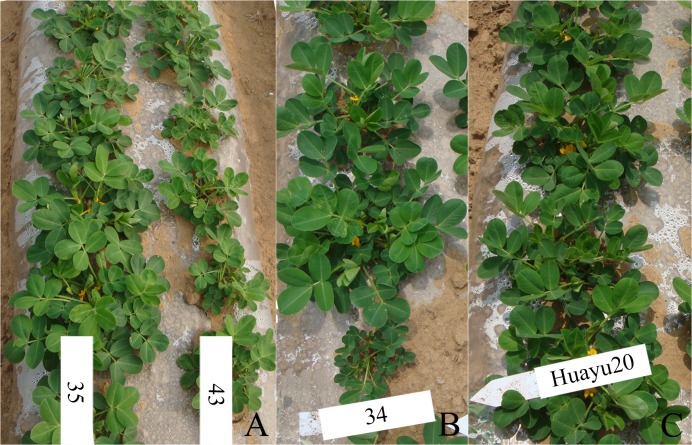
Variation and segregation among the offspring of specific plants derived from the M_2_ generation of Huayu 20 during the early growth stage. **a** Offspring of plant no. 35 and plant no. 43 differed in growth and vigor during the early growth stage; **b** Offspring of plant no. 34 differed in size; c Huayu 20, the mutagenic parent.

During early vegetative peanut production growth, offspring from different HYP-tolerant, regenerated plants differed in growth and vigor. For example, offspring of plant no. 35 were more vigorous and taller and flowered earlier than those of plant no. 43 (Fig. 2A). Some of the offspring of the HYP-tolerant, regenerated plants had large leaves. Although there was substantial variation in the offspring derived from the same plant (Fig. 2B), i.e., segregation was evident among the offspring. Seeds of mutagenic parent produced offspring that were uniform in growth and appearance (Fig. 2C).

**Fig 3:**
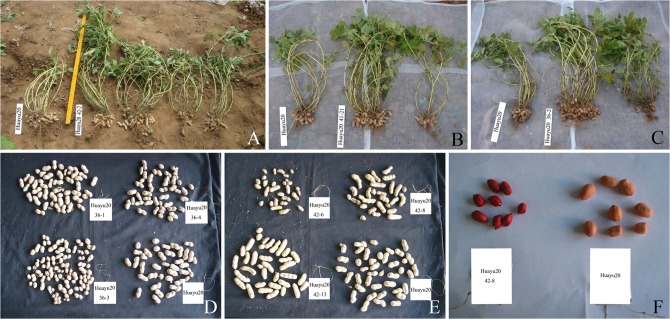
Variation and segregation among the offspring of specific plants derived from the M_2_ generation after harvest. **a** Offspring of plant no. 42 differed in plant height, flowering habit, and branch number; **b** Offspring of plant no. 41 differed in plant architecture and branch number; **c** Offspring of plant no. 36 differed in pod number; **d** Offspring of Plant no. 36 differed in pod shape; **e** Pods of Huayu 20 (the mutagenic parent, left) were normal, and pods of offspring of plant no. 42 were catenuliform with segregation in size; **f** The seed coats of plant no. 42 offspring were purple while those of Huayu 20 were pink.

At more advanced growth stages and at harvest, some of the offspring of the HYP-tolerant, regenerated plants showed variation and segregation in growth habit, flowering habit, plant height, maturity, and pod shape. For example, seven of 19 offspring of plant no. 42 had closely spaced branches with flowers arranged alternately on the stem, and some grew taller than the mutagenic parent (Fig. 3A). Offspring of plant no. 41 showed variation and segregation in the branch number, some offspring had closely spaced branches, with 22 branches in total, while other offspring of the same plant had 6 branches per plant (Fig. 3B). Offspring of plant no. 36 not only had closely spaced branches, but also showed variation in the pod number (Fig. 3C).The mutagenic parent, Huayu 20 produced erect plants with 9–11 branches and produced flowers continuously along the stem (Fig. 3A, B, C).

Variation and segregation in the pod shape were also evident among the offspring of plant no. 36; pods of one offspring (36–3) were gourd-shaped (Fig. 3D). The offspring of plant no. 42 (42–8) produced catenuliform pods containing 3–4 seeds with purple seed coat (Fig. 3E, F), while the mutagenic parent, Huayu 20 produced usual pods with pink seed coat (Fig. 3D, E, F).

### SOD and POD activities of the offspring from the HYP-tolerant, regenerated plants

SOD and POD activities were measured in the fresh leaves of 11 M3 individuals (from different HYP-tolerant, regenerated plants) that had been subjected to drought stress in the greenhouse. Eight offspring (31–1, 32–1, 34–2, 35–3, 38–3, 39–7, 40–5, and 41–12) had substantially higher SOD and POD activities than their mutagenic parents (Table 1).

**Table 1:** SOD and POD activities of Huayu 20 and M_3_ offspring of HYP-tolerant, regenerated plants. Values are means ± SE. The mutants are designated by two numbers separated by a hyphen. The first number refers to the regenerated plant first transplanted in the field. The second number refers to a specific offspring of that plant. Values are means ± SE. Means in a column followed by different letters are significantly different at *P* < 0.05.

Lines	SOD activity(U/g)	POD activity(U/g/min)
40–5	168.3±0.7a	1648.0±2.0b
41–12	166.5±1.4a	1433.0±1.0e
36–3	166.1±2.2a	957.0±5.0j
34–2	164.8±4.8a	1679.3±7.7a
38–3	163.9±0.7a	1576.7±8.1c
39–7	162. 7±1.2a	1322.3±0.3f
31–1	158.4±4.6ab	1453.3±8.1e
43–8	157.8±3.1ab	1110.7±9.8i
32–1	148.3±3.2b	1543.0±19.0d
35–3	147.3±4.3b	1254.7±4.8g
Huayu 20 (control)	133.5±6.2c	1216.0±10.0h
33–1	128.7±5.9c	1221.0±11.0h

### SSR analysis of the mutagenic parents and mutants

Thirty-eight SSRs markers were compared among the mutagenic parent, Huayu 20 and 13 M3 generation individuals derived from different HYP-tolerant, regenerated plants. Five mutants displayed polymorphism in the SSR analysis (Table 2). Signs of SSR polymorphism included a decrease in the number of amplified fragments, an increase in the number of fragments, and a change in fragment length with a frequency of 12.5, 25.0, and 62.5%, respectively (Table 2). Then, we worked out how different SSR markers can reveal the difference between mutagenic parent and mutants. PM472 could indicate the variation between two mutants while the other markers could reveal the variations of more than two mutants. For instance, PM188 was able to reveal the variation of four mutants. Despite PM48 and PM204 were sufficient enough to reveal the variations of six and seven mutants respectively, the polymorphic patterns didn’t resemble each other. When PM204 was adopted in the SSR analysis which involved Huayu 20 (the mutagenic parent) and its mutants, a decrease in the number of amplified fragments and a change in fragment length were detected. In contrast, when PM204 was replaced by PM48, the number of amplified fragments tended to increase in addition to the change in fragment length which was also observed in the case of PM204 (Table 2).

**Table 2:** Polymorphic analysis between the mutagenic parent Huayu 20 and 13 mutants by SSR.

SSR markers	Huayu 20	31–11	32–2	33–1	34–2	35–3	36–3	37–2	38–3	39–7	40–5	41–12	42–12	43–8
PM472	-	C	B	-	-	-	-	-	-	-	-	-	-	-
PM322	-	C	-	-	C	-	-	-	C	C	-	-	-	A
PM204	-	-	C	-	-	C	C	C	-	C	-	-	A	A
PM48	-	B	-	B	-	B	B	B	C	-	-	-	-	-
PM188	-	-	C	-	-	C	C	-	-	-	-	C	-	-

A: Decrease in the number of amplified fragments; B: Increase in the number of amplified fragments; C: Change in fragment length; A dash in the table indicates that the SSR was identical to that of the mutagenic parent.

### Measurement of peanut production of M_3_-generation individuals exposed to drought stress

In 2013, M_3_ seeds obtained from the individual plants in 2012 were sown in the field. The plants were watered according to standard practice until 50 days before harvest, at which time water was withheld to subject the plants to drought stress. The relative soil water content was about 24% at harvest. The plants (M_3_) were observed for agronomic traits. Some lines from the same original HYP-tolerant, regenerated plants still showed segregation. Some individuals produced more pods than the other sister individuals or the mutagenic parent. Compared with the mutagenic parent, Huayu 20, some offspring from different HYP-tolerant, regenerated plantlets still showed segregation in pod trait; some offspring produced smaller or more pods than the mutagenic parent, Huayu 20. All pods obtained from the individual plants were weighed, and the number of individual plants that produced > 40 g of pods were listed in Table 3. The results showed that some offspring of 10 of the 13 original HYP-tolerant, regenerated plants produced more pods (> 40 g/plant) than the mutagenic parent, Huayu 20 (33.4 g/plant). This was particularly true for the 20 offspring from the four original HYP-tolerant, regenerated plants (two from no. 31, 12 from no. 36, four from no. 37, and two from no. 41); these offspring produced >60 g pods/plant (Table 3).

**Table 3:** Number of M_3_ generation individuals (derived from the HYP-tolerant, regenerated plants of Huayu20) that produced ≥ 40.0 g of total pod weight per plant.

Identity of the original HYP-tolerant, regenerated plant	Number of M_3_ generation individuals that produced the indicated pod weight per plant				
	40.0–44.9 g	45.0–49.9 g	50.0–54.9 g	55.0–59.9 g	≥60.0 g
31	2	5	5	2	2
33	4		2		
34	1	1	3		
35		2		2	
36	3	3		7	12
37	2	1		1	4
39	1	1			
40	1				
41	3	5	3	4	2
43	1	2			

Note: The average total pod weight per plant was 33.4 g for Huayu 20.

### Analysis of variation range on quality characteristics of M_4_-generation seeds

**Fig 4:**
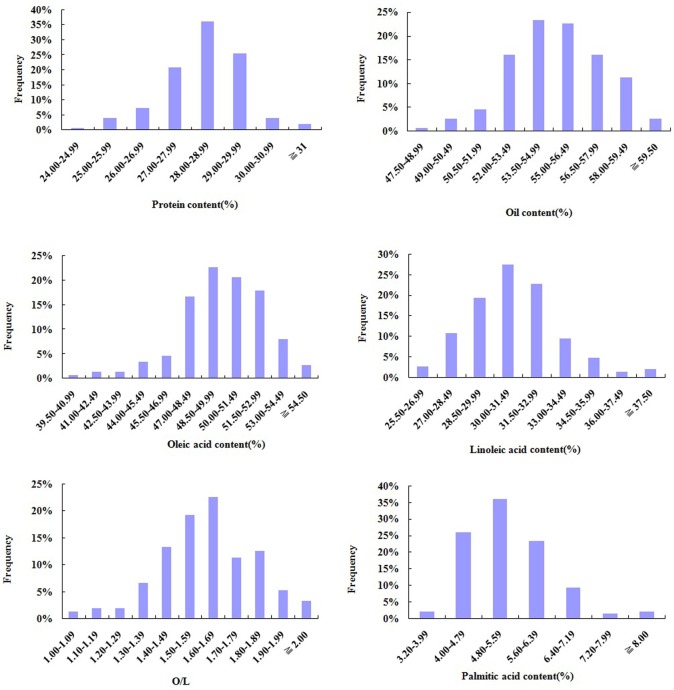
Diagram of frequency distribution on quality characteristics of M_4_-generation seeds. The contents of protein, oil, oleic acid, linoleic acid, palmitic acid and the value of oleic acid/ linoleic acid (O/L) of Huayu 20 was 26.62%, 53.78%, 49.38%,31.44%, 4.83% and 1.57, respectively. In total, M4-generation seeds from 150 M3-generation mutagenic individuals were used for calculation of quality characteristics.

The M_4_-generation seeds selected from 150 M3-generation individuals after PYM-mutagenesis and HYP-directed screening were tested for quality characteristics including contents of protein, oleic acid, linoleic acid, palmitic acid and oil by Near Infrared Spectroscopy (NIS). The result showed that variation range of protein content, oil content, palmitic acid content, oleic acid content, linoleic acid content and the ratio of oleic acid content to linoleic acid content (O/L) of M_4_-generation seeds were 24.57–31.88%, 47.68–61.10%, 3.38–10.51%, 39.86–54.99%, 25.95–39.71%, 1.00–2.12, respectively. Nine offspring with higher protein content (>30%) and 21 offspring with higher oil content (>58%) of 150 M3-generation individuals were screened (Fig. 4, Table 4).

**Table 4:** Variation range on quality characteristics of M_4_-generation seeds.

Quality characteristics of seeds	Huayu 20 (CK)	Highestvalue of mutants	Increase value	The percentage increase	Lowest value of mutants	Decrease value	The percentage decrease
Protein content (%)	26.62±0.27	31.88±0.22	5.26	19.76	24.57±0.53	−2.05	−7.70
Oil content (%)	53.79±0.96	61.10±0.84	7.31	13.59	47.68±0.99	−6.11	−11.36
Oleic acid content (%)	49.38±1.26	54.99±1.77	5.61	11.36	39.86±0.47	−9.52	−19.28
Linoleic acid content (%)	31.44±0.88	39.71±0.63	8.27	26.30	25.95±0.82	−5.49	−17.46
O/L	1.57±0.08	2.12±0.11	0.55	35.03	1.00±0.03	−0.57	−36.31
Palmitic acid content (%)	4.83±1.03	10.51±0.09	5.68	117.60	3.38±0.03	−1.45	−30.02

The pod weight per plant and main quality characteristics of the 20 M3-generation offspring of the original HYP-tolerant, regenerated plants that produced > 60 g of pods were listed in Table 5. The result showed that the oil content was substantially higher for the seeds from 19 plants than for seeds from the mutagenic parent, and the palmitic acid content and protein content were substantially higher in seeds of some offspring.

**Table 5:** Pod weight per plant and main quality characteristics of some M_4_-generation offspring that produced >60 g pods/plant.

Identity of the original HYP-tolerant, regenerated plants	Identity of offspring of the original HYP-tolerant, regenerated plants	Oil content (%)	Protein content (%)	Palmitic acid content (%)
HY20	HY20	53.79±0.96	26.62±0.27	4.83±1.03
31	31–3–1	54.70±1.92	27.96±0.46	6.16±0.10
31	31–6–7	57.80±0.54**	27.76±0.49	5.69±0.41
36	36–1–4	57.13±0.93**	27.90±0.67	5.47±0.79
36	36–2–4	58.84±0.33**	25.70±0.20	4.28±0.48
36	36–3–1	58.44±0.82**	25.93±0.26	4.34±0.46
36	36–3–2	61.10±0.84**	25.09±0.42	5.76±0.39
36	36–4–2	58.23±0.39**	26.68±0.01	3.80±0.60
36	36–4–3	60.42±0.94**	24.57±0.53*	4.22±0.34
36	36–4–4	58.76±0.07**	26.62±0.00	5.96±0.23
36	36–6–1	56.12±0.27*	28.42±0.06	7.26±0.34**
36	36–6–2	58.26±0.14**	26.62±0.37	5.26±1.11
36	36–6–3	57.61±0.10**	27.57±0.10	4.33±0.61
36	36–6–4	59.11±0.62**	25.59±0.37	4.43±0.25
36	36–6–5	55.72±0.87*	29.33±0.47**	5.23±0.41
37	37–2–1	56.45±0.17**	28.79±0.01*	7.18±0.08**
37	37–4–2	57.88±0.31**	27.91±0.61	5.05±0.63
37	37–4–3	59.27±1.62**	26.46±0.80	6.10±0.98
37	37–4–4	58.85±0.05**	26.58±0.62	6.45±0.72*
41	41–21–8	57.90±0.61**	27.80±0.26	5.44±0.12
41	41–21–18	56.58±0.64**	27.81±0.71	5.80±1.03

Value are mean ± SD, *and** are significantly different at *P* < 0.01and *P* < 0.05, respectively, the same as following tables.

To further examine the quality of HYP-tolerant mutants, we selected nine lines of the M_4_-generation individuals with higher oil content and Huayu 20 to test their oil content by NIS method and residue method as instructed by the Ministry of Agriculture of P.R.China (NY/T1285-2007). The results showed that there was no significant difference in the oil content of these nine individuals and Huayu 20 by the methods (Table 6).

**Table 6:** Result comparasion of oil content of some M_5_-generation individuals with higher oil content between NIS method and residue method.

Identity of offspring of the original HYP-tolerant, regenerated plants	Oil content (%)	
	NIS method	Residue method
HY20	50.95±0.90	49.50±0.11
36–1–4–1	57.92±0.59	57.23±0.13
36–3–2–2	56.80±0.41	56.47±0.11
36–6–1–6	58.58±0.30	57.56±0.10
36–6–4–1	57.72±0.44	58.34±0.11
37–2–1–1	57.59±0.51	57.73±0.16
37–4–4–1	58.70±1.49	57.23±0.13
37–4–4–4	58.24±0.00	56.68±0.18
41–21–18–4	59.76±1.00	58.45±0.07
41–21–18–6	59.79±0.35	59.48±0.23

## Discussion

With the increase in agricultural land that is subject to drought, the development of high-yielding and drought-tolerant cultivars has become an important breeding objective. Mutagenesis can produce new variants with new traits which are not found in the nature and can thus enrich the genetic and breeding resources available for drought resistance and other desirable traits [7,21,22]. The antibiotic PYM as a mutagen has direct mutagenic effects on DNA, it can make the DNA break at high concentration, and interfere DNA replication and synthesis at low concentration by inhabiting enzyme activity of DNA polymerases and ligases [23], which is being widely used in crop development because of its safety and high efficiency [24]. In wheat, when a PYM solution was injected into leaf veins and apical meristems, the mutant offspring differed in terms of ear length, maturity, grain number per spike, weight per 1000 grains, plant height, and other traits [25,26]. In another study, mutants of chrysanthemum with variations in leaf color, leaf type, and flower color and pattern were generated after PYM was added to the induction medium used for *in vitro* culture [27]. In our previous study, in which PYM was added to the induction medium to generate somatic embryos of peanut from embryonic leaflets, the frequency of somatic embryo formation decreased significantly with the increase in the concentration of PYM, and the suitable mutagenic concentration was determined to be 3–4 mg/L [10,13]. Based on that result, embryonic leaflets from mature peanut seeds (Huayu 20) were cultuered on a somatic embryogenesis-induction medium containing 4 mg/L PYM in the current study.

For creation and screening of new germplasm resources, the combination of *in vitro* mutagenesis and directed screening for mutants via tissue culture can save labor and other resources [28]. Hydroxyproline (HYP) was used for directed screening in the current study. As an osmolyte, proline plays an important role in salt tolerance, drought resistance, and other physiological processes. The proline analog HYP has been used in tissue culture to select for stress-resistant cell lines of many crops [29–31]. For example, researchers used somaclonal variations to screen HYP-tolerant mutants and finally to obtain regenerated plants with increased cold resistance [31]. Drought-tolerant and cold-resistant mutants of *Medicago sativa* were obtained via *in vitro* mutagenesis and directed screening with HYP, and POD activity was greater in the mutants than in the mutagenic parent [30]. In our study, the explants with somatic embryos that survived on the medium supplemented with PYM were transferred to a somatic embryo germination medium containing HYP for HYP-tolerant screening. To date, the regeneration potential of peanut was mainly restricted by its strong root failure in regeneration. In this study, we resorted to the grafting method to resolve this problem rather than the traditional tissue culture rooting method. By comparison, seedlings from the grafting method were more thickset and needed a shorter acclimation period than those generated from the rooting method [1]. The 13 HYP-tolerant, regenerated plants (the M_1_ generation) produced pods after grafting and were transplanted to the field. The pods produced by each plant were sown in the field in the following year (the M_2_ generation). Eleven offspring of the 13 HYP-tolerant, regenerated plants differed from their mutagenic parents in various characteristics including seedling vigor, plant shape, plant height, flowering traits, and pod shape. In addition, trait segregation was evident.

The activities of POD and SOD in plants have become important indices in stress-related breeding. By scavenging oxygen free radicals, POD can reduce tissue damage, increase resistance to stress, and delay senescence. POD activity in plant cells is closely related to stress tolerance [30]. SOD can scavenge superoxide anion radicals and thereby reduce the damage to membranes caused by reactive oxygen species or other peroxide free radicals [32]. The SOD activity was higher in drought-tolerant groundnut cultivars than in drought-susceptible cultivars under severe drought stress [32]. When *in vitro* mutagenesis and HYP selection pressure were used to generate stress-resistant mutants in cabbage, the regenerated plants that were HYP tolerant had increased drought resistance and higher activities of SOD and POD than the mutagenic parent [29].

After the HYP-tolerant seedlings that were grown from M_3_ generation seeds in the greenhouse were subjected to drought stress in our study, POD and SOD activities in leaves were measured. The results showed that SOD and POD activities were substantially higher for offspring of eight HYP-tolerant, regenerated plants than for the mutagenic parents.

In order to test whether gene mutation plays a role in plant trait changes, 13 M3 generation individuals of different HYP-tolerant, regenerated plants were selected for SSR analysis. By comparing the specific bands detected with those of the mutagenic parent, SSR analysis revealed that most of these plants were geneticallt different from their mutagenic parent plants. Further research is required if we plan to find out whether and how SSR markers are correlated with specific traits of HYP-tolerant plants.

After the plants had been exposed to drought stress in the field for 50 days before harvest, some M3 generation individuals from HYP-tolerant, regenerated plants of Huayu 20 produced greater total pod weight per plant than the mutagenic, Huayu 20 (33.4 g/plant), and 20 offspring produced substantially greater total pod weight (>60 g/plant). Moreover, the oil content was substantially higher in the seeds from 19 individuals of 20 than that in seeds from the mutagenic parent, Huayu 20.

Our results demonstrate that the combination of PYM (as a mutagen) and directed in vitro screening with HYP is a promising way to create new HYP-tolerant peanut lines. The HYP-tolerant mutants obtained may be useful breeding material for developing new cultivars with high yield, oil content and drought tolerance.
